# Real-world unexpected outcomes predict city-level mood states and risk-taking behavior

**DOI:** 10.1371/journal.pone.0206923

**Published:** 2018-11-28

**Authors:** A. Ross Otto, Johannes C. Eichstaedt

**Affiliations:** 1 Department of Psychology, McGill University, Montréal, Québec, Canada; 2 Positive Psychology Center, University of Pennsylvania, Philadelphia, Pennsylvania, United States of America; University of Zurich, Switzerland, SWITZERLAND

## Abstract

Fluctuations in mood states are driven by unpredictable outcomes in daily life but also appear to drive consequential behaviors such as risk-taking. However, our understanding of the relationships between unexpected outcomes, mood, and risk-taking behavior has relied primarily upon constrained and artificial laboratory settings. Here we examine, using naturalistic datasets, how real-world unexpected outcomes predict mood state changes observable at the level of a city, in turn predicting changes in gambling behavior. By analyzing day-to-day mood language extracted from 5.2 million location-specific and public Twitter posts or ‘tweets’, we examine how real-world ‘prediction errors’—local outcomes that deviate positively from expectations—predict day-to-day mood states observable at the level of a city. These mood states in turn predicted increased per-person lottery gambling rates, revealing how interplay between prediction errors, moods, and risky decision-making unfolds in the real world. Our results underscore how social media and naturalistic datasets can uniquely allow us to understand consequential psychological phenomena.

## Introduction

Moods fluctuate considerably from day to day—often in response to events in daily life [1,2]—and these affective states exert consequential effects upon cognition that manifest in consequential behaviors such as risk-taking [3–6], possibly because they engender unrealistic expectations that positive outcomes will occur in the future [7–9]. At the same time, a body of work reveals how the impact of affectively valenced outcomes on an individual’s mood state is nuanced: positive and negative outcomes exert stronger effects on mood states when they are unexpected rather than expected [10–13]. Indeed, there are large-scale behavioral consequences of these presumed mood state fluctuations. For example, when unpredictable real-world outcomes deviate positively from expectations, increases in population risk-taking behavior are observable in day-to-day lottery gambling levels at the level of a large city [14].

While laboratory study reveals how these *prediction errors*—the difference between actual versus expected outcomes thought to play a critical role in the dopaminergic system and in learning the affective structure of the environment [15,16]—can influence momentary subjective well-being [10–12], it has yet to be demonstrated that day-to-day deviations from expectations can predict fluctuations in affective states observable in the real world. Indeed, psychologists and economists emphasize the importance of and challenges inherent in understanding shifts in population-level subjective well-being on the basis of changing real-world circumstances [17,18]. Here, we provide a novel, large-scale real-world examination of how unexpected positive outcomes can, after careful treatment of important nuisance variables, predict measurable real-world affective state changes in several large urban areas.

The difficulties inherent in measuring population-level mood states in the real world have, up to now, posed challenges to the real-world study of how emotionally valenced outcomes influence affective states. However, the availability of large geo-located social media language datasets (such as public Twitter posts or ‘Tweets’) affords a powerful method for measuring population-level mood states [19]. To this end, a line of research has developed automated techniques to estimate the emotional valence (“sentiment”) of written expressions (e.g.,[20]). These sentiment analysis techniques have revealed how mood states vary both over time [21] and, using geospatially “tagged” Tweets, across different metropolitan areas [22].

In turn, researchers have successfully leveraged these inferred city- and community-level psychological states to understand and predict deleterious public health outcomes [23] and public opinion [24,25]. These advances in tracking psychological variables through social media datasets using methods of natural language processing and machine learning (including sentiment analysis) afford an unprecedented opportunity to measure day-to-day fluctuations in mood states at the level of a city. Here, by leveraging the size as well as temporal and geographic specificity of Twitter-expressed sentiment across a number of U.S. cities, we examine whether prediction errors stemming from affectively valenced and unpredictable events—previously observed to explain fluctuations in laboratory-assessed mood states [12] and real-world risk-taking levels [14]—predict day-to-day variability in Twitter-inferred city-level mood states.

A second challenge in predicting city-level mood states is pinpointing affectively valenced real-world events that generate prediction errors over time. Conveniently, sports and sunshine outcomes occur outside the control of individuals but can exert pervasive and measureable effects on mood states [4,26–29]. More specifically, the outcomes of games played by local professional sports teams and the amount of visible sunlight yield sequences of outcomes that produce useful day-to-day timecourses of prediction errors, calculated simply as positive (or negative) deviations from short-term historical trends [14]. It should be noted that these outcomes—and the prediction errors they engender—differ conceptually from the ‘reward prediction errors’ previously demonstrated in laboratory studies to drive mood state changes [12] insofar as they are not intrinsically rewarding outcomes but rather sources of valenced incidental information. Here we examine whether these prediction errors can predict observable changes in population-level mood states assessed via social media. We make the assumption that the effects of the outcomes operate on the level of the day (as opposed to say, the month or year) on the basis of a body of work which consistently demonstrates the predictive power of weather and sports outcomes at this level of temporal specificity [4,14,30].

Further, if shifts in mood state can drive changes in risk-taking behavior, as suggested by laboratory-based research [3–5,31] and the observation that naturalistic prediction errors predict shifts in real-world gambling levels [14], then measured population-level mood state fluctuations—either spontaneous or explained by local outcomes—should predict day-to-day fluctuations in risk-taking. Importantly, the popularity and widespread availability of state lottery gambling yield large datasets that afford the statistical power necessary to detect subtle changes in risk attitudes. We hypothesized that positive prediction errors stemming from local sports and sunshine outcomes should drive detectable, positive changes in Twitter-inferred mood states, and these city-level mood states should in turn engender local increases in risk-taking behavior as measured by per-person lottery gambling rates.

Accordingly, we analyzed local sports- and sunshine-based prediction errors and Twitter-inferred mood states in 27 counties spanning 6 metropolitan statistical areas (MSAs). Then, in a subset of these MSAs, we examined if 1) these inferred citywide mood states predict increased lottery gambling rates and 2) the previously observed relationships between local prediction errors and increased gambling rates are mediated by inferred citywide mood states. In keeping with best statistical practices for analysis of observational data, we separate our exploratory dataset (the year 2012 and gambling only in New York City), which guided our data analysis strategy and inferential tests, from our confirmatory dataset (the year 2013 and gambling in both New York City and Chicago), for which results and inferential tests are reported. Further, our confirmatory analyses, guided by the exploratory dataset, examine real-world lottery gambling data in a separate, additional urban area, demonstrating the geographic generality of the relationships in question here and providing assurance against spurious and/or non-replicable results[32].

## Methods

Our data sources and statistical procedures are described in detail below. Readers seeking an intuitive understanding of logic guiding our analyses and datasets are encouraged to advance to the Results section.

### Lottery data

We acquired daily lottery purchases, by postal code, for the years 2012 (exploratory dataset) and 2013 (confirmatory dataset) from the NYS Gaming Commission using a Freedom Of Information Act (FOIA) request. We aggregated daily lottery ticket sales, across 174 postal Codes, for all daily, non-jackpot-based lottery games available in New York State. Through a separate FOIA request, we acquired daily lottery purchases, for all postal codes in the Chicago MSA for the year 2013 (our confirmatory dataset; all exploratory data analyses were conducted on New York City data) from the Illinois Lottery. We aggregated the daily lottery ticket sales, across these 210 postal codes, for all daily, non-jackpot-based lottery games available in Illinois.

Because jackpot amounts are not publicly disclosed before daily drawings, and winning odds remain constant (in all of these games, prizes are awarded to players whose chosen numbers match the drawn numbers regardless of the number of winning players), the expected values of each of these games (payoff × probability of winning) remain constant over days. For each postal code, we summed the sales of these games and divided this composite by the postal code’s adult population to control for population differences across postal codes yielding a measure of per capita purchases per day [33].

### Twitter data

We used Tweets from Twitter’s freely available feed which consists of a random sample of 1% of Tweets, and used the location field listed in the users’ profile to automatically determine the presumed county of origin of the Tweet, following previous work^*21*^. To estimate the valence and arousal of Tweets, we used information a two-step process of model building and model application. The model we used was built to estimate the emotional valence and arousal of Facebook status messages, accomplished by first having multiple human raters perform manual annotation of 2,895 Facebook status messages [34]. The text of these messages was encoded as statistical distributions of language features using the open-source Python-based Differential Language Analysis ToolKit [35] (DLATK; see dlatk.wwbp.org). Specifically, we extracted: (a) the relative frequencies of words and phrases; (b) 2,000 Latent Dirichlet Allocation (LDA) topics derived in previous work^*33*^ and (c) the relatives frequencies of use in Linguistic Inquiry and Word Count dictionaries [36] (LIWC) (LDA topics are clusters of semantically coherent words, produced by a process akin to factor analysis but more appropriate for the distributional properties of words. See reference [37] for an introduction). Using the rater’s annotations as ground-truth, a (machine-learning based) ridge prediction model was trained (in DLATK) to predict the valence of the Facebook statuses. Specifically, Principal Component Analysis (PCA) was used to reduce the dimensionality of the feature space (down from 10s of thousands of features to 1,439 components) to guard against over-fitting. The performance of the model was evaluated using 10-fold cross-validation, with an out-of-sample cross-validated prediction accuracy of *r* = .65, which may be interpreted as the model’s reliability.

In the model application step, we extracted the same set of language features for the sample of random Tweets, and applied our prediction model to estimate affective valence of the Tweets. We restricted the counties to the 6 MSAs examined here, resulting in a corpus of 12.2 million Tweets across 2012 and 2013. In this way, we used a prediction model to recreate the annotators’ judgement across a large sample of Tweets. As an additional source of validation, we compared our valence estimates to those generated by an established Twitter sentiment model (SwissCheese [38]) over a subsample of 2.6 million Tweets, obtaining decent congruence (correlation of *r =* .*52;* see S2 Fig), We then averaged the estimated valence of the individual Tweets for a given day and county to obtain daily mood estimates for the counties.

### Sports outcome data

We obtained outcomes (wins, losses, and ties) of regular and post-season games played by all National Football League (NFL), National Basketball League (NBA), National Hockey League (NHL), and Major League Baseball (MLB) based in the 6 MSAs considered here, from the website Sports Reference (www.sports-reference.com).

For each team we constructed a daily, exponentially weighted average of team success:

$$P_{win}(t+1)=P_{win}(t)+\alpha [O(t)-P_{win}(t)]$$

where *t* is the day of the year, *O*(*t*) is the outcome (win = 1, loss = 0, tie = 0.5) on that day, and *α* is a recency parameter (i.e., learning rate) that makes outcomes in more recent days more influential than those in earlier days. This exponential averaging model is broadly used in behavioral and neural analyses of this sort [10,39]. The *α* parameter was set to a value of 0.1 for all analyses, a learning rate for which there is strong behavioral evidence [14,40]. On days where a team did not play, *P*_*win*_ was simply carried forward from the previous day, making our analysis of prediction error analogous to the trial-based learning algorithms used in the experimental literature [12]. The Prediction Error (*PE*) for a team on a given day is calculated as the difference between that day’s expected outcome *P*_*win*_(*t*)—the moving average from the previous day—and the outcome that day, *O*(*t*):

$$PE(t)=O(t)-P_{win}(t)$$

**Fig 1:**
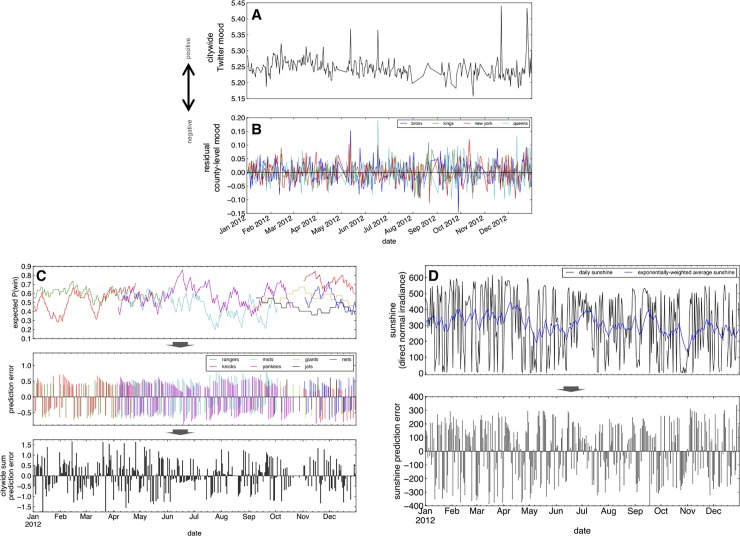
Timecourse of Twitter-inferred mood. (A) The timecourse of Twitter-inferred mood of NYC residents exhibits marked day-to-day variability. (B) Controlling for a number of nuisance variables, fluctuations in mood appear to be prominent and correlate across counties (i.e., boroughs) within NYC. (C) Exponentially-weighted estimates of winning probabilities of each NYC-based sports team based on recent outcome history (top panel). Each time a team plays, a prediction error is computed as the deviation between the outcome (win versus loss) and the expected probability of winning (middle panel). The citywide sports prediction error (bottom panel) was computed by summing each team’s prediction error for each day, reflecting a citywide deviation from expectation amongst teams that played each day. (D) From day-to-day satellite-derived sunshine levels (black line, top panel) in NYC, we calculated an exponentially-weighted expectation of sunshine level (blue line) and in turn, computed a daily sunshine prediction error based on the deviation between current and expected sunshine levels (bottom panel). All data plotted in this figure are from the exploratory dataset (the year 2012).

On each day, the PEs resulting from teams that played on that day were summed to compute a citywide sports PE (Fig 1C).

This same computation was applied for all NFL (National Football League), NBA (National Basketball League), NHL (National Hockey League), and MLB (Major League Basketball) games in the 5 other MSAs in question (see below), resulting in citywide sports PEs for Los Angeles, Chicago, Dallas-Fort Worth, the San Francisco Bay Area, and Boston. These PEs were subsequently entered into regressions to assess their impact on lottery gambling rates and Twitter-assessed mood.

### Solar irradiance data

We used satellite-derived estimates of Direct Normal Irradiance (DNI), a measure of solar irradiance in units of *W*/*m*^2^ on a surface normal to the sun, obtained on public license from Clean Power Research (www.SolarAnywhere.com). Larger DNI values indicate clearer sky conditions (i.e., sunshine). These hourly, satellite-derived irradiance estimates are demonstrated to accord with surface-based irradiance measurements [41]. For each day in 2012 and 2013 we computed the mean non-zero (hours between sunset and sunrise yield estimates of zero) DNI, which served as our daily estimate of solar irradiance. From each daily mean, we constructed a daily exponentially weighted average, computed analogously to the sports indices described above:

$$\bar{DNI}(t+1)=\bar{DNI}(t)+\alpha [DNI(t)-\bar{DNI}(t)]$$

As above, *α* was set to a value of 0.1, and the PE for a given day was calculated as the difference between *DNI*(*t*) and $\bar{DNI}(t)$. This same computation was applied to DNI data for the 5 other MSAs in question (see below), resulting in citywide sunshine PEs for Los Angeles, Chicago, Dallas-Fort Worth, the San Francisco Bay Area, and Boston. These PEs were then related to gambling behavior and Twitter-assessed mood on the same day.

#### Demographic data

From the US Census Bureau’s American Community Survey 2012 estimates, we obtained the number of residents for all counties considered in the Twitter sentiment analyses, and within NYC and Chicago, the number of adult residents in each postal code. These populations were used as sample weights in the sentiment and lottery purchase rate regressions described below so that the regression model treats more populous counties (in the case of the sentiment-based analyses, which was conducted at the county level) and more populous postal codes (in the case of the NYC and Chicago lottery gambling analyses, which are conducted at the postal level) as more representative of each population in question [42]. Further, these postal code adult populations were used to construct the per capita gambling measure described above.

### Nuisance variables

Because of cyclicality inherent to data series of this sort, we specified a number of dummy variables to control for day-of-week effects, holidays, common paycheck cycles, and severe weather events. We constructed individual dummy-coded regressors for all seven days of the week and the months of the year, and, following prior work[43], dummy-coded regressors for U.S. national holidays that fall on Mondays or Fridays (Presidents' Day, Martin Luther King Jr. Day, St. Valentine’s Day, Memorial Day, Labor Day, Columbus Day, Veterans’ Day), as well as the following days: Jan 1 and 2, Easter Sunday, July 4, Thanksgiving Day, and Christmas Day.

In the regression models with lottery purchase rate as the outcome variable, we also attempted to capture potential lottery gambling behavior stemming from income receipt using separate dummy-coded for common paycheck receipt days on day 1 and 15 of each the month (if these fell on weekends, the immediately preceding weekday was used) [14,43]. Using historical data obtained from weatherunderground.com, we constructed a regressor coding for Hurricane Sandy (Oct 29-Nov 1, 2012) in New York City as well as a regressor coding for blizzards in both New York City and Chicago, defined as days where both snow occurred and average visibility was below 5 miles (2 days in 2012 and 6 days in 2013 in New York and 7 days in 2013 in Chicago).

### Selection of U.S. MSAs, counties, and postal codes

Following previous work *[14]*, to ensure that the MSAs considered would contain adequate temporal coverage in terms of sports outcomes and contained multiple counties that exhibited sufficient Twitter activity (described below), we selected MSAs that corresponded to the five next largest media market sizes *[44]* after NYC which are also home to three or more teams across the MLB, NFL, NBA, and NHL: Los Angeles, Chicago, Dallas-Fort Worth, the San Francisco Bay Area, and Boston.

As aggregated county-level Twitter data characteristically entails sporadic missing data, we sought to identify and analyze only the counties for which there was sufficient and consistent amounts of Twitter data. Accordingly, from each MSA, we excluded counties for which there were fewer than 80% of days (out of the calendar year, for each dataset in question) where at least 100 Tweets were recorded. This ensured that calendar year coverage of Twitter sentiment data was roughly similar to sports- and sunshine-based prediction errors. This exclusion criteria left 9 counties in the NYC MSA, 4 counties in the San Francisco Bay Area MSA, 4 counties in the Los Angeles MSA, 2 counties in the Chicago MSA, 4 counties in the Boston MSA, and 4 counties in the Dallas Fort-Worth MSA. For the analyses of lottery gambling in New York City and Chicago, we excluded postal codes for which there were fewer than 15,000 residents to ensure that lottery purchase rates reflect the behavior of an informative sample.

The analyses examining lottery gambling were limited to postal codes belonging to counties that met the selection criteria above, ensuring that each postal code’s lottery gambling time course contained sufficient Twitter sentiment data to assess the relationships of interest. Accordingly, the NYC- and Chicago-based MSAs contained 163 and 137 postal codes, respectively.

### Regression models

Linear regressions—both with Twitter-inferred mood as an outcome variable and as a predictor variable—were conducted as mixed-effects models, performed using the lme4 package[45] in the R programming language. The linear model included a series of dummy-coded nuisance repressors specified above. The predictors of interest were entered into the regression as z-scores (for each analysis, separate models were estimated for sports and sunshine-related prediction errors). Regressions estimating the effect of prediction errors upon Twitter-inferred mood were performed using the entire 2013 dataset with 6 MSAs (each MSA had a unique time course of prediction errors corresponding to its local outcomes) with nested random effects taken over the MSA level and the county level, which was estimated using 9,018 total observations. Importantly, any MSA-level or county-level baseline differences in Twitter-inferred mood were accounted for by the random intercept terms in the model. Separate regressions estimating the effect of Twitter-inferred mood upon lottery gambling were performed on NYC (59,492 observations) and Chicago (76,452 observations). Coefficient estimates and statistics are reported as fixed effects at the population level in all Supporting Tables (S1–S8 Tables). Model *R*^*2*^ values are reported as conditional coefficient of determination, computed using the MuMIn package in R[46].

To quantify the average causal mediation effect of Twitter-assessed mood, we performed model-based mediation analyses in the confirmatory dataset, using the ‘mediation’ package for R[47]. This method takes as input a fitted mediator model (a mixed-effects regression with mood as outcome and either sports- or sunshine-based prediction errors as predictor variables) and a fitted outcome model (a mixed-effects regression with log per-capita lottery purchase rates as the outcome variable and either sports- or sunshine-based prediction errors and mood as predictor variables), and returns an estimate of the Average Causal Mediation Effect—that is, the proportion of the relationship between prediction errors and lottery gambling that is mediated by mood. This effect is estimated by performing 10,000 simulations using a quasi-Bayesian Monte Carlo method based on normal approximation (the default for the ‘mediation’ package).

#### Data sharing

We have made sports and sunshine data, lottery purchase rates, county-level sentiment estimates, the IDs of the Tweets analyzed by us available on a public Open Science Framework repository (accessible at http://osf.io/pd2gj). Online data collection procedures complied with terms of services for web-based sources used here.

## Results

### Estimation and timecourse of citywide mood

We first obtained a random sample of 5.2 million Tweets geo-tagged to one of 27 counties belonging to six major US metropolitan statistical areas (MSAs) of interest—New York City, Chicago, San Francisco Bay Area, Boston, Dallas Fort-Worth, and Los Angeles—based upon the locations the users have listed in their Twitter profiles (see [23]). We then used a previously derived and validated language-based prediction model [34] to estimate the affective valence of every Tweet, which we then averaged for a given day within each county to obtain daily mood estimates for each county.

To illustrate, Fig 1A depicts a timecourse of citywide composite of Twitter-inferred mood state in NYC in our exploratory dataset (2012). We removed the influence of day-of-week, month-of-year, and a number of other nuisance factors from each county’s timecourse using linear regression to eliminate cyclical and seasonal sources of variability (the timecourse of four counties are plotted in Fig 1B). The inter-correlated residual Twitter mood timecourses between these NYC counties (mean *r* = 0.19) and between counties within MSAs (mean *r* = 0.23 across MSAs) suggests that the same locally relevant outcomes potentially drive mood changes across different regions of a metropolitan area.

### Sports-based prediction errors and citywide mood

After obtaining timecourses of game outcomes for all major professional sports teams based in each MSA, we calculated a historical expectation of winning by exponentially-weighted averaging the timecourse of wins and losses (Fig 1C). On each day a team played, we calculated the discrepancy between the game’s outcome and the expectation that day, yielding a timecourse of prediction errors—positive when a team performs better than expected and negative when a team performs worse than expected—that spans the team’s playing season. This Reinforcement Learning-based formalization of prediction errors is widespread in behavioral and neurobiological accounts of human choice behavior[10,39]. Aggregating these timecourses across all teams in an MSA yielded a “citywide sum sports prediction error” that spanned the entire calendar year which parsimoniously captures how much better or worse the city’s sports teams performed, as a whole, relative to short-term historical expectations.

**Fig 2:**
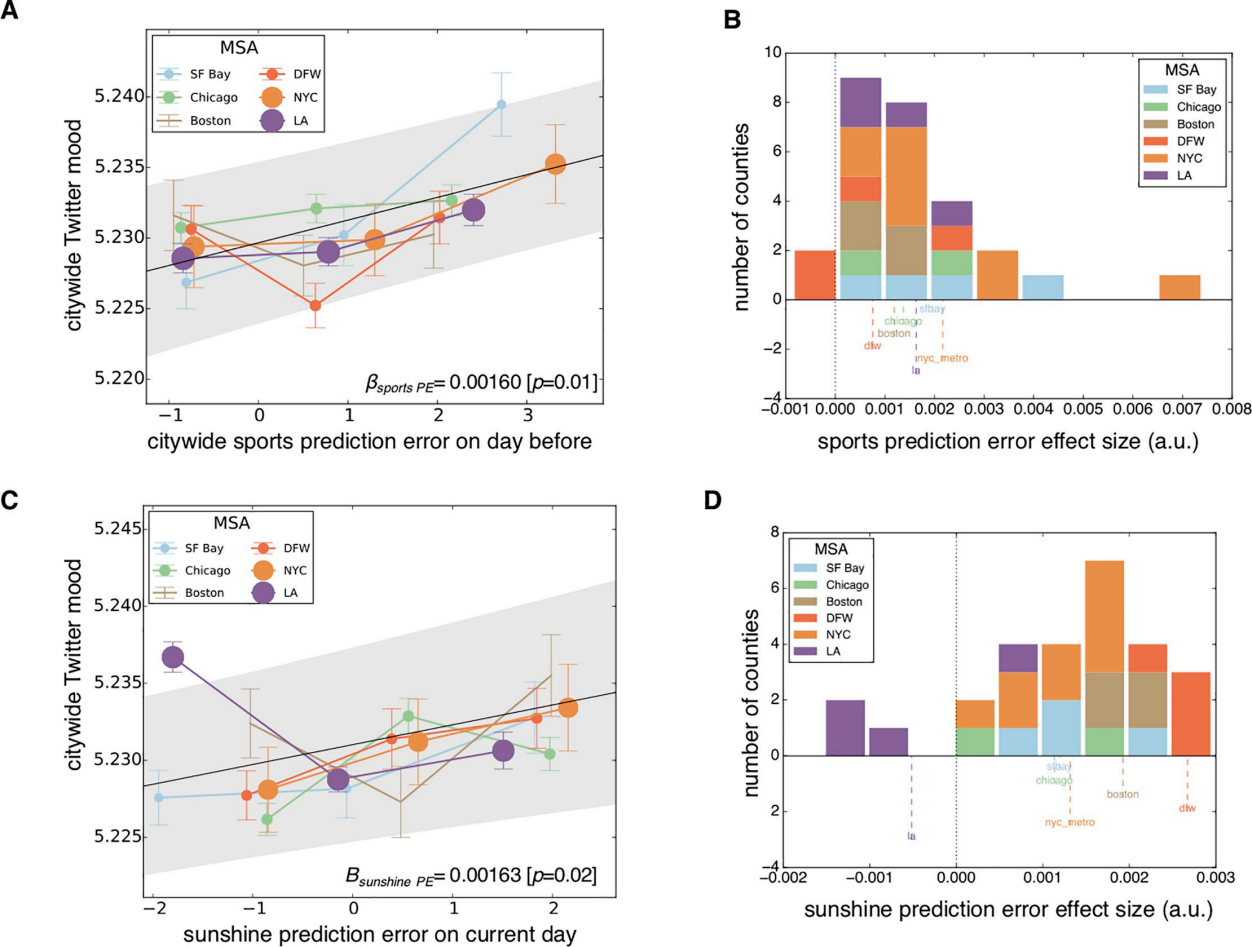
Sports prediction errors and Twitter-inferred mood. (A) When the aggregate of local sports teams performs better than expected on the previous day—engendering a positive citywide sports prediction error—Twitter-inferred citywide mood increases significantly. Residual Twitter-inferred mood is plotted for all Metropolitan Statistical Areas (MSAs), after controlling for a number of nuisance variables, along the vertical axis. The populations of each MSA included are represented by point size. The black solid line represents the regression line corresponding to the effect of sports prediction errors across all MSAs (shaded gray represents standard error) and the dashed line represents a zero-magnitude effect or baseline). (B) Histogram of effect sizes for citywide sports prediction errors for each of across the 27 counties examined (color indicates the MSA to which each county belongs). The county-level effect size distribution, obtained from this regression model, suggests that the positive effect of local sports prediction errors on subsequent county-level mood is nearly unanimous. (C) When sunshine levels are higher than expected based on recent history, Twitter-inferred citywide mood increases significantly on the present day. (D) The distribution of county-level sunshine prediction-error effects are generally positive with the apparent exception of counties in the Los Angeles MSA. All data plotted in this figure are from the confirmatory dataset (the year 2013).

We were thus positioned to analyze how MSA-level prediction errors could predict mood state fluctuations in each MSA’s constituent counties. As sports outcomes appear to exert the largest behavioral impact on the day following the sporting event [14,26], we estimated the effect of sports prediction errors upon city-level mood on the next day. Compellingly, the exploratory dataset suggested a roughly linear predictive relationship between sports prediction errors and residual city-level mood (S1 Fig). We then tested this relationship in our confirmatory dataset, finding that when local sports teams performed better than expected, city residents expressed significantly more positive affect on social media (Fig 2A). This relationship was confirmed, statistically, with mixed-effects regression, finding a modest predictive effect (*β* = 0.00160, *p* = 0.01; *R*^*2*^ = 0.301; see S1 Table for full regression coefficient estimates). Further, the distribution of county-level effect sizes, grouped by MSA (Fig 2B), reveals a marked consistency across MSAs of the positive effect of these prediction errors upon mood states, suggesting that the relationship between prediction errors and mood states generalizes across geographic regions.

## Sunshine-based prediction errors and citywide mood

To examine how prediction errors stemming from sunshine could also impact city-wide mood states, we similarly calculated sunshine prediction errors by computing the deviation between satellite-derived estimates of solar irradiance each day from an exponentially-weighted expectation of sunshine, yielding a timecourse of sunshine prediction errors (an example NYC timecourse is plotted in Fig 1D). Intuitively, this prediction error is positive when a sunny day follows a streak of cloudy days and negative when a cloudy day follows a streak of sunny days.

Across the same six urban areas, we found a suggestive relationship between sunshine prediction and city-level mood on the current day in our exploratory dataset (S1 Fig), which we then verified in our confirmatory dataset (Fig 2C, *β* = 0.00163, *p* = 0.023; *R*^*2*^ = 0.284; see S2 Table for full regression coefficient estimates). These results indicate that unexpected weather outcomes exhibit similar positive predictive effects upon citywide mood as sports. The county-level effect size distributions reveal how the majority of these MSAs respond positively to unexpectedly sunny days (Fig 2D). Perhaps unsurprisingly, Los Angeles—which exhibited the least variability in solar irradiance in the calendar year of all MSAs in question (*SD*: 92.64 *W*/*m*^2^) exhibited no observable positive mood reactivity to sunshine prediction errors (by contrast, the *SD* of the Dallas-Fort-Worth area was 128.08 *W*/*m*^2^). Intuitively, the moods of individuals in a region with little day-to-day variability in sunshine levels do not appear to respond to these weather-related prediction errors.

Importantly, we found no effect of either prediction error source on overall level of Tweeting (measured as the number of Tweets/day/county) with respect to either sports (*β* = 4.25, *p* = .73) or sunshine (*β* = 1.112, *p* = .88) prediction errors, suggesting that these unexpected outcomes were specific to changes in affective content in Tweets and not individuals’ overall amount of Twitter expression.

### Prediction errors, citywide mood and lottery gambling

We next turned to analyzing the timecourses of day-to-day lottery purchases across neighborhoods in NYC and Chicago—MSAs for which we were able to obtain daily lottery gambling purchasing data—to evaluate the hypothesis that these apparent population-level mood fluctuations predict changes in risk preferences. In particular, we examined per-capita purchase rates for lottery tickets with fixed payoffs and winning odds (and thus constant expected values), which serves as a proxy for changes in day-to-day risk preferences over time[48,49].

**Fig 3:**
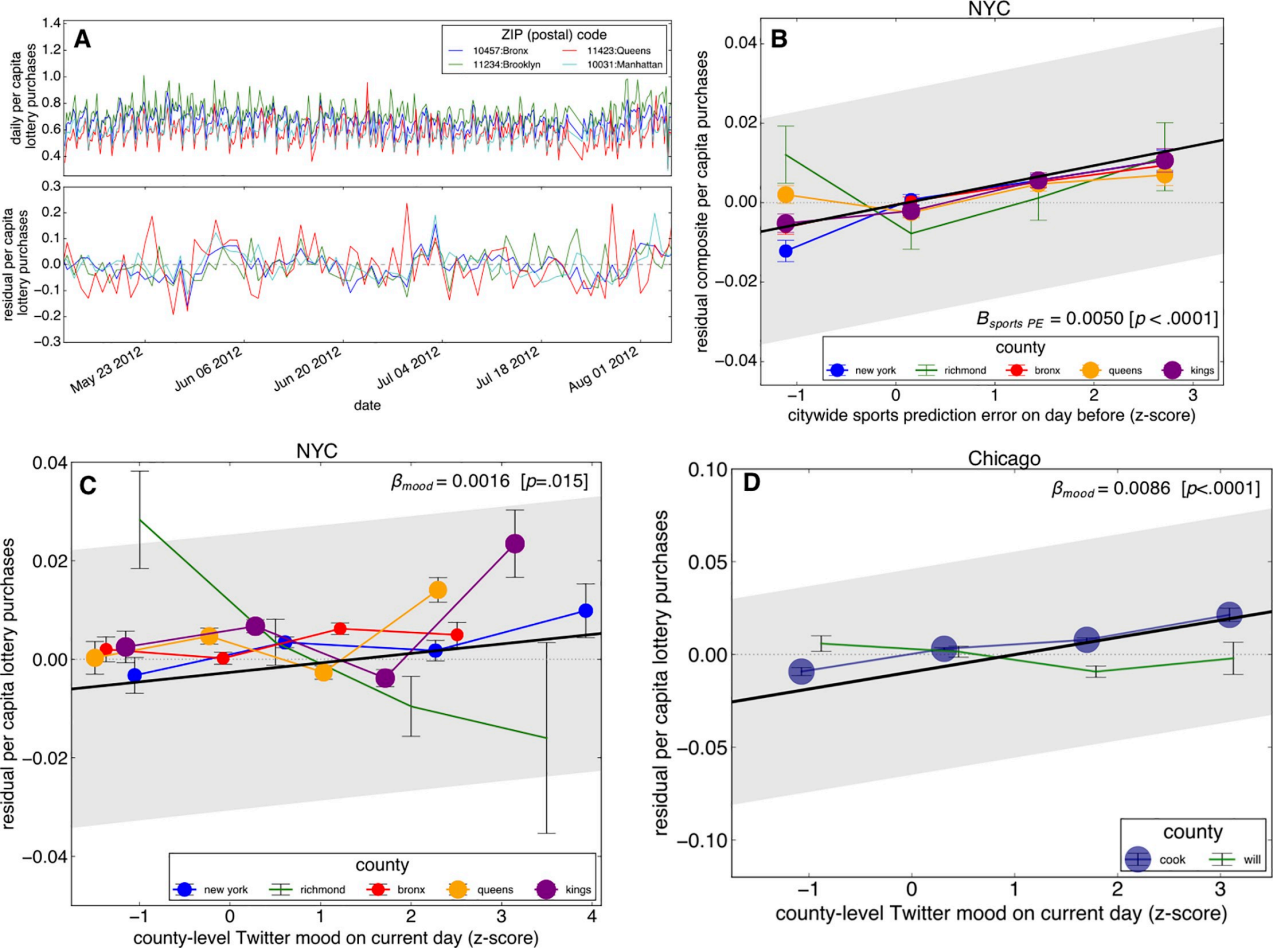
Prediction errors, lottery gambling, and twitter-inferred mood. (A) The composite per-capita purchases of daily lottery gambling in NYC exhibits substantial day-to-day variability in 2012. After controlling for nuisance variables, we still find prominent fluctuations in gambling apparently correlated at the neighborhood-level. (B) Positive citywide sports prediction errors (horizontal axis) predict increasing per-person gambling levels (plotted as residuals, after controlling for a number of nuisance variables, along the vertical axis) in NYC. (C and D) Twitter-inferred mood significantly and positively predicts per-person lottery gambling rates on the same day in the NYC and Chicago MSAs. The population of each county included in the MSA-level regression models are represented by point size. MSA-level regression lines are depicted in black. All data plotted in this figure are from the confirmatory datasets (the year 2013).

Accordingly, we analyzed timecourse data from state lotteries for the two MSAs examined here, and found that, like Twitter-inferred mood state, these daily per-capita lottery purchase rates (in USD/person) fluctuate considerably from day to day (Fig 3A) and moreover, and, after controlling for a number of nuisance variables, appear to fluctuate similarly across neighborhoods (Fig 3B), suggesting a common source of day-to-day variability in risk preference. Replicating previous work[14], we found in our confirmatory dataset that both sports- and sunshine-based prediction errors predicted increased per-person lottery gambling rates in both NYC and Chicago (Fig 3B; S5–S8 Tables), suggesting again that prediction-error driven perturbations in mood states could explain these changes in real-world risk-taking levels.

We then examined if Twitter-inferred mood states might exert predictive bearing on per capita lottery gambling rates, suggestive positive relationships in most counties in our exploratory dataset (NYC in the year 2012, S1 Fig). We then confirmed these relationships statistically in our confirmatory datasets (NYC and Chicago in 2013, finding modest but statistically significant relationships between mood state and same-day per capita lottery gambling in both NYC (Fig 3C; *β* = 0.0019; *p* = 0.015; *R*^*2*^ = 0.0781) and Chicago (Fig 3D; *β* = 0.0093; *p*<0.0001; *R*^*2*^ = 0.0393; S3 and S4 Tables). In other words, on days when residents of these cities expressed more positive moods on Twitter—which itself appears to be driven by prediction errors—residents of these cities engaged in more risk-taking behavior.

**Fig 4:**
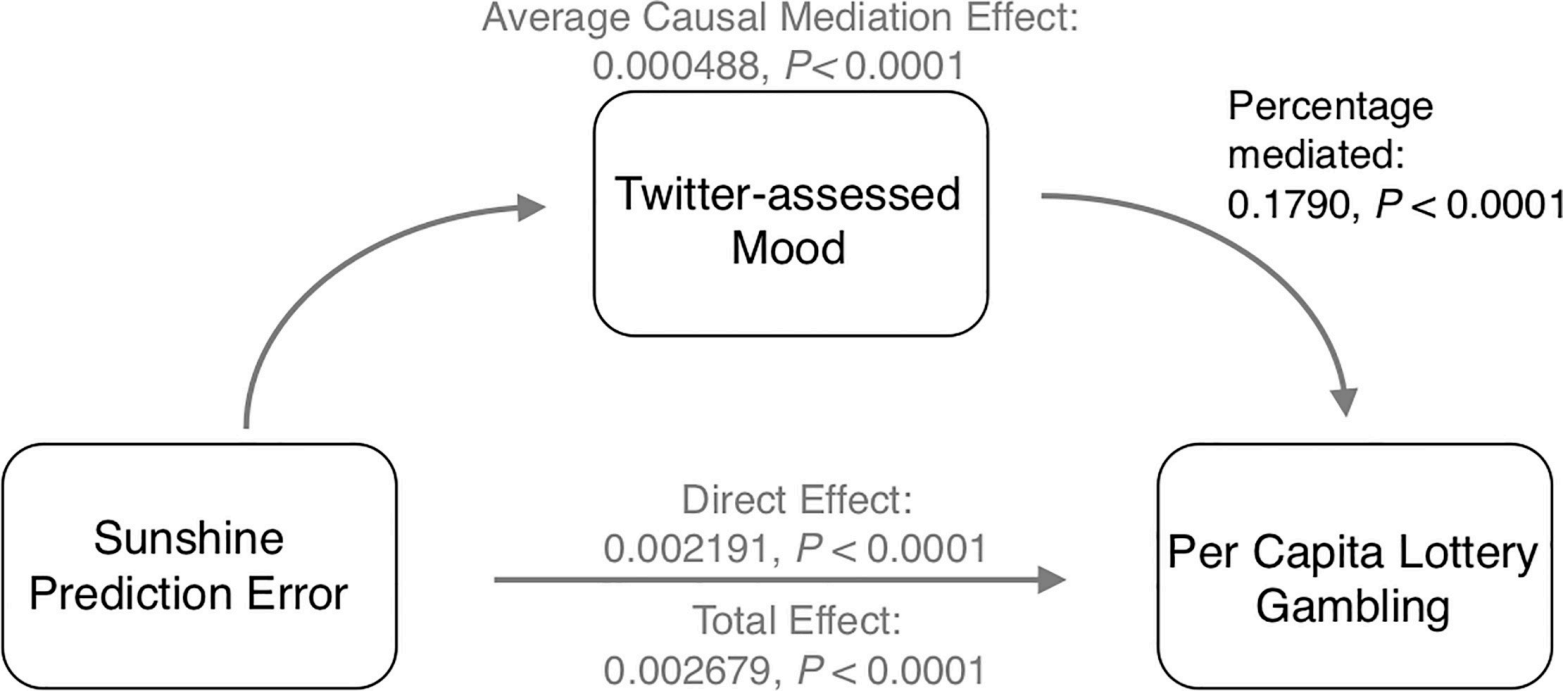
Mediation model. Diagram of model used to investigate the extent to which Twitter-assessed mood mediates the relationship between sunshine prediction errors in Chicago (Confirmatory dataset).

Finally, we tested whether the observed relationships between prediction errors and risk-taking are mediated, statistically, by Twitter-inferred mood state. In the Chicago dataset, we found a significant mediation effect in sports (*p* = .04, based on a proportion mediated of 7.46% based on a quasi-Bayesian Monte Carlo method) and sunshine prediction errors (*p*<0.0001; proportion mediated of 17.9%), supporting the notion that these unexpected positive outcomes foster risk-taking in part through changes in mood (S9 and S10 Tables; see example mediation model diagram in Fig 4). However, in the NYC dataset, we found partial support for this sort of mediation: Twitter-inferred mood significantly mediated the relationship between sports-based prediction errors and lottery gambling (*p* = 0.04; S11 Table), but did not significantly mediate the relationship between sunshine-based prediction errors and lottery gambling (*p* = 0.34; S12 Table).

## Discussion

Leveraging a large-scale dataset of social media sentiment, we reveal how observable city-level mood fluctuations can be explained by prediction errors stemming from affectively valenced and locally germane events across several large urban areas. Indeed, the ability to predict city-level mood changes on the basis of these unexpected positive and negative outcomes—despite the multi-causal nature of aggregate mood states and inherent noisiness of social-media-inferred expression of mood—underlines the influence of prediction errors upon mood states which has previously only been established in laboratory settings[10–12].

In turn, the predictive relationship between day-to-day measured mood state fluctuations and real-world risk preferences complements an existing body of work investigating the malleability of human risk preferences grounded in traditional psychological methods[3,4,50,51]. One theory posits that positive affect fosters feelings of optimism, which biases an individual’s perception of the likelihood of positive events occurring [7,8,52] while another account posits that positive moods engender a favorable perception of the outcomes of risky choices [5]. Intriguingly, the observed relationship between city-level mood state and gambling is compatible with both accounts, underscoring how large-scale real-world investigations of this sort lend external validity to psychological theories rooted in laboratory-based research.

The finding that citywide mood states can be explained by prediction errors further highlights the usefulness of large-scale behavioral studies for understanding psychological questions[53]. At the same time, laboratory study of the linkage between mood and gambling still faces two potential limitations. First, consequential manipulations of mood (i.e., involving valenced, real-world outcomes) are pragmatically challenging to carry out, and second, and the laboratory gambling tasks used to study risk taking are often artificial and/or hypothetical measures which do not necessarily relate to real-world risk taking behaviours[54]. In the present study, the ubiquity of affectively valenced prediction errors stemming from locally relevant outcomes and the size and richness of social media language datasets[19] permit examination of the impact of collective events upon subjective well-being at the scale of large cities. Likewise, large naturalistic datasets like state lotteries provide a proxy variable for population-wide shifts in preferences to engage in risk-taking behavior [49]. Further, these results dovetail with laboratory-based examination of the relationship between prediction errors and mood states (as demonstrated previously[12]) but also compellingly suggest that these momentary mood state changes evoked by prediction errors stemming from risky choice outcomes could, in a carefully controlled laboratory design, detectably shift subsequent risk preferences in the same choice setting.

Although prediction error effects upon inferred mood states are subtle, they are comparable in magnitude to season-evoked mood changes[21], and the generality of these prediction error effects in both domain and geography is noteworthy. Still, it is unlikely that these observed effects of sunshine prediction errors merely reflect seasonal or daylength-based effects on mood state as 1) the timescale at which these prediction errors are being calculated based on short-term history and 2) seasonal variations such as the month of the year are captured as nuisance variables in the regression model and thus, the estimated effects of prediction errors reflect variation in mood unexplained by simple seasonal variations. Our analysis is based on a prior body of work presuming that weather-based outcomes exert influence over affective states on the timescale of a single day[4] and the day after in the case of sports[4,14,26]. However, future work is need to determine if these sorts of real-world unexpected outcomes appear to predict changes in affective states or risk-taking levels on longer timescales. Relatedly, the prediction errors considered in this study are calculated from outcome expectations based upon the recent outcome histories rather other sources of expectations such as weather forecasts in the case of sunshine, or betting the relative strengths of the teams in the case of sports. The extent to which deviations from these qualitatively different sources of expectations can also account for fluctuations in mood state and risk-taking remains an important, but unexplored question.

While this analysis explains how city-level changes in mood state are driven, in part, by real-world outcomes, and further, elucidates the real-world behavioral consequences of mood states, a potential limitation of these datasets stems from the possibility that Twitter users may not be representative of lottery gamblers (and vice versa). In particular, Twitter users tend to be younger and more educated [55] while lottery gamblers tend to be less educated and older than the general population[48]. Still, the predictive relationship between sentiment expressed on social media and gambling suggest that these are reasonable proxy variables for mood states and risk-taking behaviors, respectively, which manifest at the city level. Relatedly, future work should investigate whether the observed effects manifest 1) outside of US population centers, and 2) populations outside of Western, educated, industrialized, rich and democratic (WEIRD) countries[56].

Nonetheless, these predicted per-person changes in gambling behavior (Fig 3) are economically consequential at the community level as we found that a high level of positive Twitter-inferred mood (2 SDs above the mean) predicted an increase in spending of 1.9 cents per person per day on lottery gambling in responsive Chicago neighborhoods and 1.2 cents in particularly responsive New York City neighborhoods. As previous work demonstrates that these lottery products are disproportionally purchased by low-income individuals as evidenced in our own datasets[14] and by other investigators[33,57], these expenditures can be particularly deleterious as money spent on lottery gambling has been shown to displace other useful household expenditures[58].

So-called ‘Big Data’ methods have the potential to suggest evidence-based policy interventions with unprecedented levels of contextual nuance and sensitivity[59]. For example, at present, lottery advertising is pervasive and often promotes misleading and provocative messages which foster unrealistic attitudes about the benefits of lottery play[60]—and these messages are demonstrated to sustain and/or intensify established gambling habits[61]. The present results suggest that affective predictors of gambling can be measured through social media, and could possibly be used to inform more precisely targeted interventions promoting responsible gambling. Finally, as a number of pathological behaviors (e.g., substance abuse) are thought to stem from the same aberrant psychological processes as excessive gambling[62]. Future work should evaluate the suitability of other measurable variables (for example, misdemeanor complaints putatively stemming from reckless behavior) which might also afford insight into societally and economically consequential city-level risk-taking behaviors[63].

## Supporting information

S1 Methods Regression equations. (PDF)

S1 Fig Exploratory analyses conducted on 2012 datasets. (A) Relationship between citywide sports prediction errors on the previous day and Twitter-inferred citywide mood. (B) Relationship between sunshine prediction errors on the current day and Twitter-inferred citywide mood. The population of each MSA included in the regression model is represented by point size. The black line represents the regression line corresponding to the effect of sports prediction errors across all MSAs (shaded gray represents standard error of the regression line). (C) Relationship between Twitter-inferred mood upon per-person lottery gambling rates in NYC. The population of each county included in the MSA-level regression models are represented by point size. MSA-level regression lines are depicted in back (shaded gray represents standard error).
(PDF)

S2 Fig Correspondence of our valence-predictions and SwissCheese, a standard Twitter sentiment tool (Deriu, Gonzenbach, Uzdilli, Lucchi, Luca & Jaggi, 2016). (PDF)

S1 Table Fixed-effects regression coefficients for model estimating effect of Citywide (Sum) Sports PEs upon Twitter-inferred Mood across all MSAs (2013; Confirmatory Dataset). (DOCX)

S2 Table Fixed-effects regression coefficients for model estimating effect of Sunshine PEs upon Twitter-inferred mood across all MSAs (2013; confirmatory dataset). (DOCX)

S3 Table Fixed-effects regression coefficients for model estimating effect of Twitter-inferred Mood upon log per-person lottery purchases in New York city (2013; confirmatory dataset). (DOCX)

S4 Table Fixed-effects regression coefficients for model estimating effect of Twitter-inferred mood upon log per-person lottery purchases in Chicago (2013; confirmatory dataset). (DOCX)

S5 Table Fixed-effects regression coefficients for model estimating effect of citywide sports PEs upon log per-person lottery purchases in New York City (2013; confirmatory dataset). (DOCX)

S6 Table Fixed-effects regression coefficients for model estimating effect of Sunshine PEs upon log per-person lottery purchases in New York city (2013; confirmatory dataset). (DOCX)

S7 Table Fixed-effects regression coefficients for model estimating effect of citywide sports PEs upon log per-person lottery purchases in Chicago (2013; confirmatory dataset). (DOCX)

S8 Table Fixed-effects regression coefficients for model estimating effect of Sunshine PEs upon log per-person lottery purchases in Chicago (2013; confirmatory dataset). (DOCX)

S9 Table Estimated causal effects in mediation analysis examining citywide Sports PEs (direct effect), Twitter-inferred mood (indirect effect), and Per-capita log per-person lottery purchases (outcome variable) in Chicago (2013; confirmatory dataset). (DOCX)

S10 Table Estimated causal effects in mediation analysis examining sunshine PEs (direct effect), Twitter-inferred mood (indirect effect), and Per-capita log per-person lottery purchases (outcome variable) in Chicago (2013; confirmatory dataset). (DOCX)

S11 Table Estimated causal effects in mediation analysis examining citywide Sports PEs (direct effect), Twitter-inferred mood (indirect effect), and Per-capita log per-person lottery purchases (outcome variable) in New York city (2013; confirmatory dataset). (DOCX)

S12 Table Estimated causal effects in mediation analysis examining sunshine PEs (direct effect), Twitter-inferred mood (indirect effect), and Per-capita log per-person lottery purchases (outcome variable) in New York city (2013; confirmatory dataset). (DOCX)
