# Supplementary material for: Real-world unexpected outcomes predict city-level mood states and risk-taking behavior
Source: PLoS One. 2018 Nov 28;13(11):e0206923. doi: 10.1371/journal.pone.0206923 (PMC6261541; doi:10.1371/journal.pone.0206923)
Supplement: S1 Methods — (PDF) [file pone.0206923.s001.pdf]

Supporting Information:

Real-world unexpected outcomes predict city-level mood states and risk-taking behavior

A. Ross Otto & Johannes C. Eichstaedt

Regression Equations

The model formulae as entered into the lmer() function in the lme4 package for R were as follows for estimating the effects of sports and sunshine based prediction errors, respectively, upon mood:

```
mood ~  sunshine_pe + TUE + WED + THU + FRI + SAT + SUN + FEB + MAR + APR + MAY +
JUN + JUL + AUG + SEP + OCT + NOV + INDEPENDENCEDAY + THANKSGIVING +
DAYAFTERCHRISTMAS + NEWYEARSEVE + EASTER + MEMORIALDAY + VALENTINESDAY + (1 +
sunshine_pe + TUE + WED + THU + FRI + SAT + SUN + FEB + MAR + APR + MAY + JUN + JUL
+ AUG + SEP + OCT + NOV + INDEPENDENCEDAY + THANKSGIVING + DAYAFTERCHRISTMAS +
NEWYEARSEVE + EASTER + MEMORIALDAY + VALENTINESDAY | msa/county )
```

```
mood ~  sports_pe + TUE + WED + THU + FRI + SAT + SUN + FEB + MAR + APR + MAY + JUN
+ JUL + AUG + SEP + OCT + NOV + INDEPENDENCEDAY + THANKSGIVING + DAYAFTERCHRISTMAS
+ NEWYEARSEVE + EASTER + MEMORIALDAY + VALENTINESDAY + (1 + sports_pe + TUE + WED +
THU + FRI + SAT + SUN + FEB + MAR + APR + MAY + JUN + JUL + AUG + SEP + OCT + NOV +
INDEPENDENCEDAY + THANKSGIVING + DAYAFTERCHRISTMAS + NEWYEARSEVE + EASTER +
MEMORIALDAY + VALENTINESDAY | msa/county )
```

and for estimating the effect of mood upon per capita lottery purchases:

```
log_purchase ~  mood + TUE + WED + THU + FRI + SAT + SUN + FEB + MAR + APR + MAY +
JUN + JUL + AUG + SEP + OCT + NOV + DEC + FIRST_OF_MONTH + FIFTEENTH_OF_MONTH +
STORM + INDEPENDENCEDAY + THANKSGIVING + CHRISTMASDAY + DAYAFTERCHRISTMAS +
LABORDAY + EASTER + NEWYEARSDAY + COLUMBUSDAY + MEMORIALDAY +
BIRTHDAYOFMARTINLUTHERKINGJR + VETERANSDAY + WASHINGTONSBIRTHDAY + VALENTINESDAY +
(1 + mood + TUE + WED + THU + FRI + SAT + SUN + FEB + MAR + APR + MAY + JUN + JUL +
AUG + SEP + OCT + NOV + DEC + FIRST_OF_MONTH + FIFTEENTH_OF_MONTH + STORM +
INDEPENDENCEDAY + THANKSGIVING + CHRISTMASDAY + DAYAFTERCHRISTMAS + LABORDAY +
EASTER + NEWYEARSDAY + COLUMBUSDAY + MEMORIALDAY + BIRTHDAYOFMARTINLUTHERKINGJR +
VETERANSDAY + WASHINGTONSBIRTHDAY + VALENTINESDAY | ZIP )
```
