# Supplementary material for: Real-world unexpected outcomes predict city-level mood states and risk-taking behavior
Source: PLoS One. 2018 Nov 28;13(11):e0206923. doi: 10.1371/journal.pone.0206923 (PMC6261541; doi:10.1371/journal.pone.0206923)

S1 Figure. Exploratory analyses conducted on 2012 datasets. (A) Relationship between citywide sports prediction errors on the previous day and Twitter-inferred citywide mood. (B) Relationship between sunshine prediction errors on the current day and Twitter-inferred citywide mood. The population of each MSA included in the regression model is represented by point size. The black line represents the regression line corresponding to the effect of sports prediction errors across all MSAs (shaded gray represents standard error of the regression line). (C) Relationship between Twitter-inferred mood upon per-person lottery gambling rates in NYC. The population of each county included in the MSA-level regression models are represented by point size. MSA-level regression lines are depicted in back (shaded gray represents standard error).

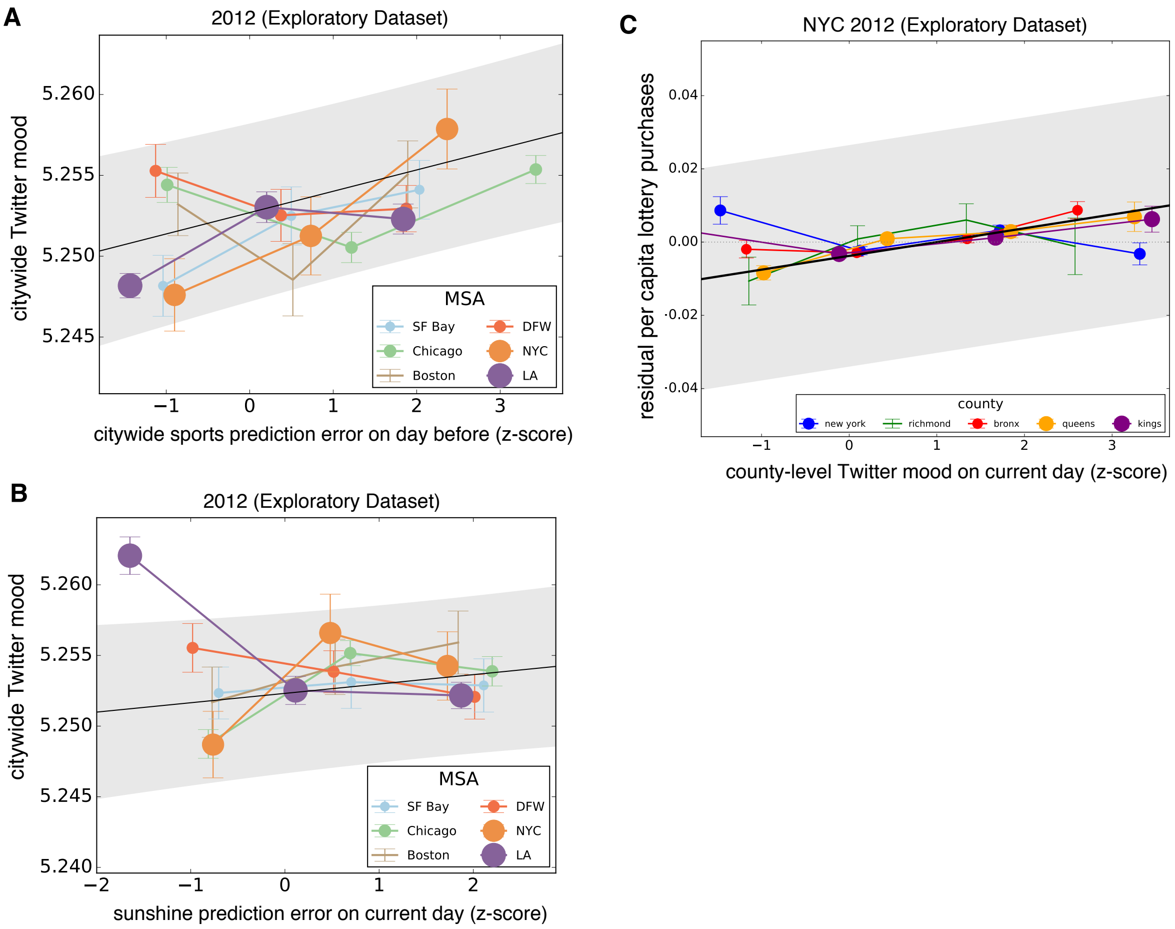

Supplement: S1 Fig — (A) Relationship between citywide sports prediction errors on the previous day and Twitter-inferred citywide mood. (B) Relationship between sunshine prediction errors on the current day and Twitter-inferred citywide mood. The population of each MSA included in the regression model is represented by point size. The black line represents the regression line corresponding to the effect of sports prediction errors across all MSAs (shaded gray represents standard error of the regression line). (C) Relationship between Twitter-inferred mood upon per-person lottery gambling rates in NYC. The population of each county included in the MSA-level regression models are represented by point size. MSA-level regression lines are depicted in back (shaded gray represents standard error). (PDF) [file pone.0206923.s002.pdf]
