# Supplementary material for: Real-world unexpected outcomes predict city-level mood states and risk-taking behavior
Source: PLoS One. 2018 Nov 28;13(11):e0206923. doi: 10.1371/journal.pone.0206923 (PMC6261541; doi:10.1371/journal.pone.0206923)

S2 Figure. Correspondence of our valence-predictions and SwissCheese, a standard Twitter sentiment tool (Deriu, Gonzenbach, Uzdilli, Lucchi, Luca & Jaggi, 2016).

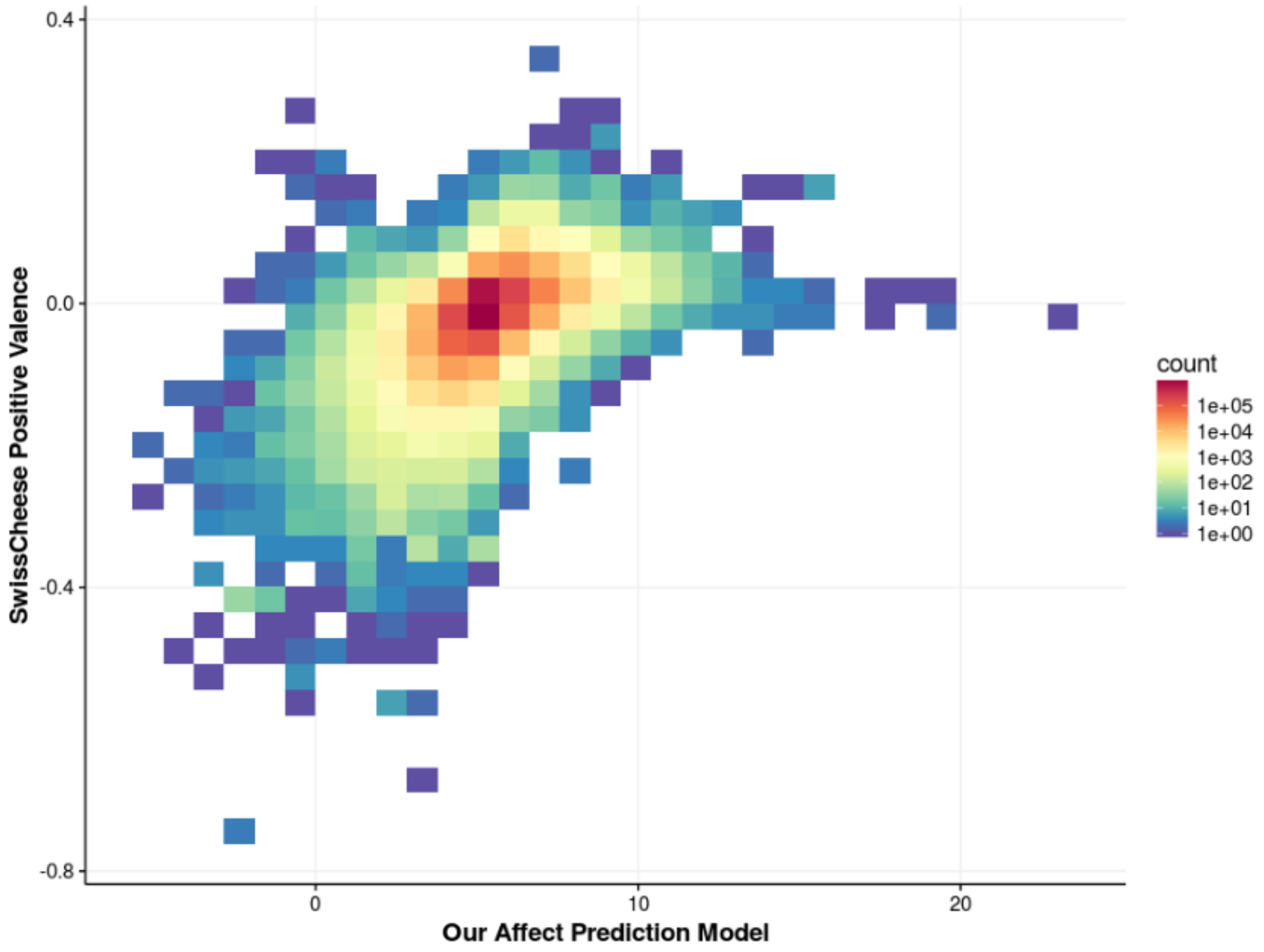

Supplement: S2 Fig — (PDF) [file pone.0206923.s003.pdf]
