# Supplementary material for: Real-world unexpected outcomes predict city-level mood states and risk-taking behavior
Source: PLoS One. 2018 Nov 28;13(11):e0206923. doi: 10.1371/journal.pone.0206923 (PMC6261541; doi:10.1371/journal.pone.0206923)
Supplement: S1 Table — (DOCX) [file pone.0206923.s004.docx]

S1 Table. Fixed-effects regression coefficients for model estimating effect of Citywide (Sum) Sports PEs upon Twitter-inferred Mood across all MSAs (2013; Confirmatory Dataset).

| *Coefficient* | *Estimate (SE)* | *p-value* |
| --- | --- | --- |
| (Intercept) | 5.2158 (0.0068) | <.0001* |
| **Citywide Sports PE** | **0.0016 (0.0006)** | **0.010*** |
| TUE | -0.0048 (0.0034) | 0.162 |
| WED | -0.0079 (0.0033) | 0.016* |
| THU | -0.0021 (0.0032) | 0.526 |
| FRI | 0.0169 (0.0034) | <.0001* |
| SAT | 0.0179 (0.0037) | <.0001* |
| SUN | 0.0122 (0.0033) | <.0001* |
| JAN | 0.0205 (0.0036) | <.0001* |
| FEB | 0.0031 (0.0037) | 0.409 |
| MAR | 0.0090 (0.0042) | 0.030* |
| APR | -0.0072 (0.0050) | 0.155 |
| MAY | 0.0084 (0.0035) | 0.017* |
| JUN | 0.0105 (0.0043) | 0.014* |
| JUL | -0.0022 (0.0050) | 0.663 |
| AUG | 0.0064 (0.0048) | 0.185 |
| SEP | 0.0087 (0.0030) | 0.004* |
| OCT | 0.0073 (0.0061) | 0.233 |
| NOV | 0.0095 (0.0030) | 0.001* |
| INDEPENDENCEDAY | 0.0189 (0.0058) | 0.001* |
| THANKSGIVING | -0.0008 (0.0052) | 0.877 |
| DAYAFTERCHRISTMAS | 0.0312 (0.0133) | 0.019* |
| NEWYEARSEVE | 0.1846 (0.0177) | <.0001* |
| EASTER | 0.0523 (0.0174) | 0.003* |
| MEMORIALDAY | 0.0665 (0.0274) | 0.015* |
| VALENTINESDAY | 0.0374 (0.0106) | <.0001* |
