# Supplementary material for: Real-world unexpected outcomes predict city-level mood states and risk-taking behavior
Source: PLoS One. 2018 Nov 28;13(11):e0206923. doi: 10.1371/journal.pone.0206923 (PMC6261541; doi:10.1371/journal.pone.0206923)
Supplement: S2 Table — (DOCX) [file pone.0206923.s005.docx]

S2 Table. Fixed-effects regression coefficients for model estimating effect of Sunshine PEs upon Twitter-inferred Mood across all MSAs (2013; Confirmatory Dataset).

| *Coefficient* | *Estimate (SE)* | *p-value* |
| --- | --- | --- |
| (Intercept) | 5.2210 (0.0058) | <.0001* |
| **Sunshine PE** | **0.0016 (0.0007)** | **0.023*** |
| TUE | -0.0053 (0.0027) | 0.052 |
| WED | -0.0017 (0.0035) | 0.628 |
| THU | -0.0003 (0.0028) | 0.908 |
| FRI | 0.0200 (0.0034) | <.0001* |
| SAT | 0.0185 (0.0033) | <.0001* |
| SUN | 0.0121 (0.0029) | <.0001* |
| JAN | 0.0116 (0.0036) | 0.001* |
| FEB | -0.0053 (0.0054) | 0.323 |
| MAR | 0.0006 (0.0058) | 0.921 |
| APR | -0.0152 (0.0109) | 0.163 |
| MAY | 0.0001 (0.0046) | 0.974 |
| JUN | 0.0023 (0.0059) | 0.691 |
| JUL | -0.0089 (0.0063) | 0.160 |
| AUG | -0.0019 (0.0052) | 0.716 |
| SEP | -0.0003 (0.0048) | 0.953 |
| OCT | -0.0035 (0.0072) | 0.629 |
| NOV | 0.0015 (0.0047) | 0.745 |
| FIRST_OF_MONTH | 0.0159 (0.0044) | <.0001* |
| FIFTEENTH_OF_MONTH | -0.0012 (0.0049) | 0.811 |
| INDEPENDENCEDAY | 0.0271 (0.0164) | 0.100 |
| THANKSGIVING | 0.1826 (0.0163) | <.0001* |
| DAYAFTERCHRISTMAS | 0.0418 (0.0160) | 0.009* |
| NEWYEARSEVE | 0.0683 (0.0141) | <.0001* |
| EASTER | 0.0375 (0.0143) | 0.009* |
