# Supplementary material for: Real-world unexpected outcomes predict city-level mood states and risk-taking behavior
Source: PLoS One. 2018 Nov 28;13(11):e0206923. doi: 10.1371/journal.pone.0206923 (PMC6261541; doi:10.1371/journal.pone.0206923)
Supplement: S3 Table — (DOCX) [file pone.0206923.s006.docx]

S3 Table. Fixed-effects regression coefficients for model estimating effect of Twitter-inferred Mood upon log per-person lottery purchases in New York City (2013; Confirmatory Dataset).

| *Coefficient* | *Estimate (SE)* | *p-value* |
| --- | --- | --- |
| (Intercept) | -0.6705 (0.0281) | <0.0001* |
| **Twitter Mood** | **0.0019 (0.0009)** | **0.02*** |
| TUE | 0.0084 (0.0028) | 0.00* |
| WED | 0.0708 (0.0061) | <0.0001* |
| THU | 0.0921 (0.0057) | <0.0001* |
| FRI | 0.1513 (0.0044) | <0.0001* |
| SAT | 0.0239 (0.012) | 0.05* |
| SUN | -0.1888 (0.0148) | <0.0001* |
| FEB | 0.0454 (0.0036) | <0.0001* |
| MAR | 0.1135 (0.0051) | <0.0001* |
| APR | 0.1724 (0.0058) | <0.0001* |
| MAY | 0.1152 (0.0059) | <0.0001* |
| JUN | 0.1377 (0.0065) | <0.0001* |
| JUL | 0.0223 (0.0065) | 0.00* |
| AUG | 0.0613 (0.0076) | <0.0001* |
| SEP | 0.0459 (0.0065) | <0.0001* |
| OCT | 0.0684 (0.007) | <0.0001* |
| NOV | 0.0709 (0.0062) | <0.0001* |
| DEC | 0.1198 (0.0071) | <0.0001* |
| FIRST_OF_MONTH | 0.0253 (0.0038) | <0.0001* |
| FIFTEENTH_OF_MONTH | 0.0122 (0.0032) | 0.00* |
| STORM | -0.1107 (0.0053) | <0.0001* |
| INDEPENDENCEDAY | -0.1794 (0.0178) | <0.0001* |
| THANKSGIVING | -0.4133 (0.0373) | <0.0001* |
| CHRISTMASDAY | -0.7523 (0.0357) | <0.0001* |
| DAYAFTERCHRISTMAS | 0.0319 (0.0137) | 0.02* |
| LABORDAY | -0.1194 (0.0179) | <0.0001* |
| EASTER | -0.0002 (0.01) | 0.98 |
| NEWYEARSDAY | -0.3027 (0.0447) | <0.0001* |
| COLUMBUSDAY | -0.0445 (0.0107) | <0.0001* |
| MEMORIALDAY | -0.2037 (0.0157) | <0.0001* |
| BIRTHDAYOFMARTINLUTHERKINGJR | -0.0615 (0.012) | <0.0001* |
| VETERANSDAY | -0.0163 (0.0104) | 0.12 |
| WASHINGTONSBIRTHDAY | -0.074 (0.0124) | <0.0001* |
| VALENTINESDAY | 0.0113 (0.0115) | 0.32 |
