# Supplementary material for: Real-world unexpected outcomes predict city-level mood states and risk-taking behavior
Source: PLoS One. 2018 Nov 28;13(11):e0206923. doi: 10.1371/journal.pone.0206923 (PMC6261541; doi:10.1371/journal.pone.0206923)
Supplement: S4 Table — (DOCX) [file pone.0206923.s007.docx]

S4 Table. Fixed-effects regression coefficients for model estimating effect of Twitter-inferred Mood upon log per-person lottery purchases in Chicago (2013; Confirmatory Dataset).

| *Coefficient* | *Estimate (SE)* | *p-value* |
| --- | --- | --- |
| (Intercept) | -1.4547 (0.0525) | <0.0001* |
| **Twitter Mood** | **0.0093 (0.001)** | **<0.0001*** |
| TUE | -0.17 (0.0081) | <0.0001* |
| WED | -0.0644 (0.0057) | <0.0001* |
| THU | -0.1008 (0.0079) | <0.0001* |
| FRI | -0.0151 (0.007) | 0.03* |
| SAT | 0.0376 (0.0079) | <0.0001* |
| SUN | -0.3344 (0.0119) | <0.0001* |
| FEB | 0.0928 (0.0044) | <0.0001* |
| MAR | 0.0992 (0.0057) | <0.0001* |
| APR | 0.1307 (0.0065) | <0.0001* |
| MAY | 0.0284 (0.0068) | <0.0001* |
| JUN | 0.0525 (0.0071) | <0.0001* |
| JUL | 0.0305 (0.0072) | <0.0001* |
| AUG | 0.0065 (0.0071) | 0.36 |
| SEP | 0.0217 (0.0064) | 0.00* |
| OCT | 0.0173 (0.0065) | 0.01* |
| NOV | -0.0296 (0.0068) | <0.0001* |
| DEC | 0.0654 (0.0085) | <0.0001* |
| FIRST_OF_MONTH | 0.036 (0.004) | <0.0001* |
| FIFTEENTH_OF_MONTH | 0.0561 (0.0041) | <0.0001* |
| STORM | -0.0916 (0.0057) | <0.0001* |
| INDEPENDENCEDAY | -0.2327 (0.0163) | <0.0001* |
| THANKSGIVING | -0.2699 (0.0154) | <0.0001* |
| CHRISTMASDAY | -0.6968 (0.0222) | <0.0001* |
| DAYAFTERCHRISTMAS | -0.0271 (0.0109) | 0.01* |
| LABORDAY | -0.1555 (0.0148) | <0.0001* |
| EASTER | -0.1284 (0.0118) | <0.0001* |
| NEWYEARSDAY | -0.3575 (0.0157) | <0.0001* |
| COLUMBUSDAY | 0.0159 (0.0105) | 0.13 |
| MEMORIALDAY | -0.1633 (0.0141) | <0.0001* |
| BIRTHDAYOFMARTINLUTHERKINGJR | -0.0876 (0.0105) | <0.0001* |
| VETERANSDAY | -0.0669 (0.0098) | <0.0001* |
| WASHINGTONSBIRTHDAY | -0.092 (0.0113) | <0.0001* |
| VALENTINESDAY | 0.0418 (0.0109) | 0.00* |
