# Supplementary material for: Real-world unexpected outcomes predict city-level mood states and risk-taking behavior
Source: PLoS One. 2018 Nov 28;13(11):e0206923. doi: 10.1371/journal.pone.0206923 (PMC6261541; doi:10.1371/journal.pone.0206923)
Supplement: S5 Table — (DOCX) [file pone.0206923.s008.docx]

**S5 Table.** Fixed-effects regression coefficients for model estimating effect of Citywide Sports PEs upon log per-person lottery purchases in New York City (2013; Confirmatory Dataset).

| *Coefficient* | *Estimate (SE)* | *p-value* |
| --- | --- | --- |
| (Intercept) | -0.6698 (0.0286) | <0.0001* |
| **Sports PE** | **0.0049 (0.0004)** | **<0.0001*** |
| TUE | 0.0106 (0.0024) | <0.0001* |
| WED | 0.0712 (0.0056) | <0.0001* |
| THU | 0.0945 (0.0054) | <0.0001* |
| FRI | 0.15 (0.0039) | <0.0001* |
| SAT | 0.0234 (0.0113) | 0.04* |
| SUN | -0.1852 (0.0143) | <0.0001* |
| FEB | 0.0336 (0.0032) | <0.0001* |
| MAR | 0.1069 (0.0049) | <0.0001* |
| APR | 0.1273 (0.005) | <0.0001* |
| MAY | 0.1109 (0.0055) | <0.0001* |
| JUN | 0.1121 (0.006) | <0.0001* |
| JUL | 0.0252 (0.0061) | <0.0001* |
| AUG | 0.0425 (0.0062) | <0.0001* |
| SEP | 0.0393 (0.0061) | <0.0001* |
| OCT | 0.0442 (0.0062) | <0.0001* |
| NOV | 0.0828 (0.0058) | <0.0001* |
| DEC | 0.1073 (0.0065) | <0.0001* |
| FIRST_OF_MONTH | 0.0471 (0.0032) | <0.0001* |
| FIFTEENTH_OF_MONTH | 0.0196 (0.0028) | <0.0001* |
| INDEPENDENCEDAY | -0.1796 (0.0164) | <0.0001* |
| THANKSGIVING | -0.3688 (0.0183) | <0.0001* |
| DAYAFTERCHRISTMAS | 0.0411 (0.0114) | 0.00* |
| EASTER | 0.0117 (0.0089) | 0.19 |
| COLUMBUSDAY | -0.0208 (0.009) | 0.02* |
| MEMORIALDAY | -0.1954 (0.0145) | <0.0001* |
| BIRTHDAYOFMARTINLUTHERKINGJR | -0.0632 (0.0109) | <0.0001* |
| VETERANSDAY | -0.0337 (0.0088) | 0.00* |
| WASHINGTONSBIRTHDAY | -0.0719 (0.0115) | <0.0001* |
| VALENTINESDAY | 0.0289 (0.0089) | 0.00* |
