# Supplementary material for: Real-world unexpected outcomes predict city-level mood states and risk-taking behavior
Source: PLoS One. 2018 Nov 28;13(11):e0206923. doi: 10.1371/journal.pone.0206923 (PMC6261541; doi:10.1371/journal.pone.0206923)
Supplement: S6 Table — (DOCX) [file pone.0206923.s009.docx]

**S6 Table.** Fixed-effects regression coefficients for model estimating effect of Sunshine PEs upon log per-person lottery purchases in New York City (2013; Confirmatory Dataset).

| *Coefficient* | *Estimate (SE)* | *p-value* |
| --- | --- | --- |
| (Intercept) | -0.6644 (0.0223) | <0.0001* |
| **Sunshine PE** | **0.001 (0.0004)** | **0.02*** |
| TUE | 0.0032 (0.0022) | 0.14 |
| WED | 0.0637 (0.0048) | <0.0001* |
| THU | 0.0978 (0.005) | <0.0001* |
| FRI | 0.145 (0.0036) | <0.0001* |
| SAT | 0.0228 (0.0094) | 0.02* |
| SUN | -0.1886 (0.0112) | <0.0001* |
| FEB | 0.0333 (0.0028) | <0.0001* |
| MAR | 0.1045 (0.0043) | <0.0001* |
| APR | 0.1261 (0.0045) | <0.0001* |
| MAY | 0.1092 (0.0049) | <0.0001* |
| JUN | 0.1079 (0.0053) | <0.0001* |
| JUL | 0.0191 (0.0053) | 0.00* |
| AUG | 0.0405 (0.0054) | <0.0001* |
| SEP | 0.0361 (0.0053) | <0.0001* |
| OCT | 0.0514 (0.0056) | <0.0001* |
| NOV | 0.073 (0.0052) | <0.0001* |
| DEC | 0.1013 (0.0058) | <0.0001* |
| FIRST_OF_MONTH | 0.038 (0.0029) | <0.0001* |
| FIFTEENTH_OF_MONTH | 0.016 (0.0025) | <0.0001* |
| INDEPENDENCEDAY | -0.1833 (0.0129) | <0.0001* |
| THANKSGIVING | -0.3696 (0.0139) | <0.0001* |
| CHRISTMASDAY | -0.6249 (0.0147) | <0.0001* |
| DAYAFTERCHRISTMAS | 0.0329 (0.0099) | 0.00* |
| EASTER | 0.0029 (0.0087) | 0.74 |
| NEWYEARSEVE | 0.1486 (0.0085) | <0.0001* |
| COLUMBUSDAY | -0.038 (0.0086) | <0.0001* |
| MEMORIALDAY | -0.2008 (0.0116) | <0.0001* |
| BIRTHDAYOFMARTINLUTHERKINGJR | -0.0712 (0.0097) | <0.0001* |
| VETERANSDAY | -0.0249 (0.0085) | 0.00* |
| WASHINGTONSBIRTHDAY | -0.0754 (0.0101) | <0.0001* |
| VALENTINESDAY | 0.0193 (0.0088) | 0.03* |
