# Supplementary material for: Real-world unexpected outcomes predict city-level mood states and risk-taking behavior
Source: PLoS One. 2018 Nov 28;13(11):e0206923. doi: 10.1371/journal.pone.0206923 (PMC6261541; doi:10.1371/journal.pone.0206923)
Supplement: S7 Table — (DOCX) [file pone.0206923.s010.docx]

**S7 Table.** Fixed-effects regression coefficients for model estimating effect of Citywide Sports PEs upon log per-person lottery purchases in Chicago (2013; Confirmatory Dataset).

| *Coefficient* | *Estimate (SE)* | *p-value* |
| --- | --- | --- |
| **Sports PE** | **0.0068 (0.0005)** | **<0.0001*** |
| TUE | -0.232 (0.0053) | <0.0001* |
| WED | -0.1184 (0.0039) | <0.0001* |
| THU | -0.1637 (0.005) | <0.0001* |
| FRI | -0.056 (0.0047) | <0.0001* |
| SAT | 0.0391 (0.0049) | <0.0001* |
| SUN | -0.354 (0.0069) | <0.0001* |
| FEB | 0.0861 (0.0033) | <0.0001* |
| MAR | 0.1038 (0.0033) | <0.0001* |
| APR | 0.1063 (0.0034) | <0.0001* |
| MAY | 0.0332 (0.0035) | <0.0001* |
| JUN | 0.0753 (0.0037) | <0.0001* |
| JUL | 0.0206 (0.0039) | <0.0001* |
| AUG | 0.0396 (0.0038) | <0.0001* |
| SEP | 0.0479 (0.0036) | <0.0001* |
| OCT | -0.0172 (0.004) | <0.0001* |
| NOV | -0.001 (0.0044) | 0.83 |
| DEC | 0.1062 (0.0054) | <0.0001* |
| FIRST_OF_MONTH | 0.0504 (0.0036) | <0.0001* |
| FIFTEENTH_OF_MONTH | 0.0586 (0.003) | <0.0001* |
| INDEPENDENCEDAY | -0.1909 (0.0089) | <0.0001* |
| THANKSGIVING | -0.23 (0.009) | <0.0001* |
| DAYAFTERCHRISTMAS | 0.0244 (0.0084) | 0.00* |
| EASTER | -0.1245 (0.0084) | <0.0001* |
| NEWYEARSEVE | 0.0538 (0.0085) | <0.0001* |
| MEMORIALDAY | -0.1812 (0.0091) | <0.0001* |
| BIRTHDAYOFMARTINLUTHERKINGJR | -0.0921 (0.0086) | <0.0001* |
| VETERANSDAY | -0.0771 (0.0083) | <0.0001* |
| WASHINGTONSBIRTHDAY | -0.0984 (0.0085) | <0.0001* |
| VALENTINESDAY | 0.1167 (0.0085) | <0.0001* |
