# Supplementary material for: Real-world unexpected outcomes predict city-level mood states and risk-taking behavior
Source: PLoS One. 2018 Nov 28;13(11):e0206923. doi: 10.1371/journal.pone.0206923 (PMC6261541; doi:10.1371/journal.pone.0206923)
Supplement: S8 Table — (DOCX) [file pone.0206923.s011.docx]

**S8 Table.** Fixed-effects regression coefficients for model estimating effect of Sunshine PEs upon log per-person lottery purchases in Chicago (2013; Confirmatory Dataset).

| *Coefficient* | *Estimate (SE)* | *p-value* |
| --- | --- | --- |
| (Intercept) | -1.7694 (0.0188) | <0.0001* |
| **Sunshine PE** | **0.0061 (0.0005)** | **<0.0001*** |
| TUE | -0.2179 (0.0046) | <0.0001* |
| WED | -0.0999 (0.0033) | <0.0001* |
| THU | -0.141 (0.0042) | <0.0001* |
| FRI | -0.0412 (0.0041) | <0.0001* |
| SAT | 0.0515 (0.0045) | <0.0001* |
| SUN | -0.3496 (0.0065) | <0.0001* |
| FEB | 0.0618 (0.0029) | <0.0001* |
| MAR | 0.0902 (0.0027) | <0.0001* |
| APR | 0.0947 (0.0031) | <0.0001* |
| MAY | 0.0252 (0.0032) | <0.0001* |
| JUN | 0.0625 (0.0033) | <0.0001* |
| JUL | 0.0426 (0.0037) | <0.0001* |
| AUG | 0.0277 (0.0035) | <0.0001* |
| SEP | 0.0355 (0.0035) | <0.0001* |
| OCT | 0.0024 (0.0035) | 0.49 |
| NOV | -0.0175 (0.0038) | <0.0001* |
| DEC | 0.0909 (0.0049) | <0.0001* |
| FIRST_OF_MONTH | 0.0411 (0.0029) | <0.0001* |
| FIFTEENTH_OF_MONTH | 0.0679 (0.0029) | <0.0001* |
| INDEPENDENCEDAY | -0.2314 (0.0091) | <0.0001* |
| THANKSGIVING | -0.224 (0.0087) | <0.0001* |
| CHRISTMASDAY | -0.6612 (0.0096) | <0.0001* |
| DAYAFTERCHRISTMAS | 0.0258 (0.0087) | 0.00* |
| EASTER | -0.1214 (0.0088) | <0.0001* |
| NEWYEARSEVE | 0.0705 (0.0086) | <0.0001* |
| COLUMBUSDAY | 0.0711 (0.0086) | <0.0001* |
| MEMORIALDAY | -0.1497 (0.0089) | <0.0001* |
| BIRTHDAYOFMARTINLUTHERKINGJR | -0.0868 (0.0086) | <0.0001* |
| VETERANSDAY | -0.0574 (0.0086) | <0.0001* |
| WASHINGTONSBIRTHDAY | -0.0713 (0.0087) | <0.0001* |
| VALENTINESDAY | 0.1176 (0.0087) | <0.0001* |
