# Supplementary material for: Real-world unexpected outcomes predict city-level mood states and risk-taking behavior
Source: PLoS One. 2018 Nov 28;13(11):e0206923. doi: 10.1371/journal.pone.0206923 (PMC6261541; doi:10.1371/journal.pone.0206923)
Supplement: S10 Table — (DOCX) [file pone.0206923.s013.docx]

S10 Table. Estimated causal effects in mediation analysis examining Sunshine PEs (Direct Effect), Twitter-inferred mood (Indirect Effect), and Per-capita log per-person lottery purchases (Outcome Variable) in Chicago (2013; Confirmatory Dataset).

|  | *Estimate* | *95% lower CI* | *95% upper CI* | *p-value* |
| --- | --- | --- | --- | --- |
| *Average Causal Mediation Effect* | 0.000488 | 0.000367 | 0.000651 | <0.0001* |
| *Average Direct Effect* | 0.002191 | 0.001215 | 0.003147 | <0.0001* |
| *Total Effect* | 0.002679 | 0.001699 | 0.003642 | <0.0001* |
| *Prop. Mediated* | 0.17905 | 0.115972 | 0.291046 | <0.0001* |
