# Supplementary material for: Real-world unexpected outcomes predict city-level mood states and risk-taking behavior
Source: PLoS One. 2018 Nov 28;13(11):e0206923. doi: 10.1371/journal.pone.0206923 (PMC6261541; doi:10.1371/journal.pone.0206923)
Supplement: S12 Table — (DOCX) [file pone.0206923.s015.docx]

S12 Table. Estimated causal effects in mediation analysis examining Sunshine PEs (Direct Effect), Twitter-inferred mood (Indirect Effect), and Per-capita log per-person lottery purchases (Outcome Variable) in New York City (2013; Confirmatory Dataset).

|  | *Estimate* | *95% lower CI* | *95% upper CI* | *p-value* |
| --- | --- | --- | --- | --- |
| *Average Causal Mediation Effect* | 0.0000 | 0.0000 | 0.0001 | 0.3200 |
| *Average Direct Effect* | 0.0009 | 0.0001 | 0.0016 | 0.0400* |
| *Total Effect* | 0.0009 | 0.0001 | 0.0016 | 0.0200* |
| *Prop. Mediated* | 0.0209 | -0.0408 | 0.1970 | 0.3400 |
